# Serum vitamin D levels in Berliners of Turkish descent –a cross-sectional study

**DOI:** 10.1186/s12889-019-6446-5

**Published:** 2019-01-28

**Authors:** Lilian Krist, Theresa Keller, Heiko Becher, Karl-Heinz Jöckel, Martin Schlaud, Stefan N. Willich, Thomas Keil

**Affiliations:** 1 Institute for Social Medicine, Epidemiology and Health Economics, Charité-Universitätsmedizin Berlin, Luisenstr. 57, 10117 Berlin, Germany; 2 Institute for Medical Biometry and Epidemiology, University Medical Centre Hamburg-Eppendorf, Hamburg, Germany; 3 Institute for Medical Informatics, Biometry and Epidemiology (IMIBE), University Hospital Essen, Essen, Germany; 4 Department of Epidemiology and Health Monitoring, Robert Koch-Institute Berlin, Berlin, Germany

**Keywords:** Vitamin D, Vitamin D deficiency, Migrants, Turkish, Germany, Public health

## Abstract

**Background:**

Vitamin D levels may differ between migrant and non-migrant populations, especially among non-western immigrants living in a country with limited sun exposure such as Germany. This study examined serum vitamin D concentration and associated factors among Berliners with and without Turkish background.

**Methods:**

Two samples (with and without Turkish roots) were recruited in the inner city of Berlin for a cross-sectional study assessing serum vitamin D concentration. Linear regression analyses were used to examine sociodemographic, lifestyle and medical factors associated with serum vitamin D levels.

**Results:**

In the analyses, we included 537 subjects (39% men and 61% women, age 43.2 ± 12.5 (mean ± standard deviation) years) with and 112 without Turkish background (46% men and 54% women, age 46.7 ± 14.6 years). The Turkish sample had lower mean (95%-Confidence Interval) vitamin D levels than the non-Turkish sample: 22.7 nmol/L (21.5;23.9) vs 34.7 nmol/L (31.9;37.5), *p* < 0.001. In the Turkish female subgroup, veiled women had considerably lower levels than unveiled women: 14.4 nmol/L (11.5;17.3) vs 24.9 nmol/L (23.1;26.7), p < 0.001. Multivariable regression analysis revealed that among the Berliners of Turkish descent, being active less than 150 min per day, and being overweight/obese were independently associated with a lower vitamin D concentration. In the non-migrant sample besides being overweight and obese, female sex was associated with lower vitamin D concentrations.

**Conclusions:**

Serum vitamin D levels were considerably low in Berliners of Turkish descent, and especially in veiled women. Potentially modifiable factors of low vitamin D levels were high BMI and low physical activity. These findings should be considered in the development of future public health strategies for subpopulations with Turkish migration background.

**Electronic supplementary material:**

The online version of this article (10.1186/s12889-019-6446-5) contains supplementary material, which is available to authorized users.

## Background

Vitamin D is a steroid hormone with regulatory effects on the calcium and phosphate homeostasis, parathormone (PTH) and thyroxine secretion [1]. Vitamin D also affects the cardiovascular system, diabetes, the immune system, cancer, and overall mortality [2–4]. It is usually measured in serum as 25(OH)-D_3._ Levels > 75 nmol/L have been defined as vitamin D sufficiency, while 50–75 nmol/L have been considered as vitamin D insufficiency and < 50 nmol/L as vitamin D deficiency [5]. Vitamin D deficiency over a longer period can cause a softening of the bones, termed rickets in children and osteomalacia in adults. Less severe vitamin D insufficiency may lead to an insufficient mineralization of the bone due to an increase of PTH, which can cause osteoporosis and fractures, muscle weakness and muscle pain [2, 3].

The main source of vitamin D is sunlight exposure inducing vitamin D production in the skin through ultraviolet B radiation (UVBR). Only a few foods such as fatty fish or cod liver oil contain vitamin D in a relevant amount. Vitamin D can be ingested as supplement, and in some countries foods are fortified with vitamin D [6]. The main risk factor for a poor vitamin D status is therefore a lack of exposure to sunlight. This can be caused either by sun avoidance, sunscreen, clothing habits, or an indoor lifestyle. Latitude, season and time of the day influence the UVBR [6].

Vitamin D has become a worldwide research issue and vitamin D deficiency has been described as pandemic by several authors; the lowest population-based vitamin D levels have been assessed in Asian countries such as China, Mongolia, India and the Middle East [6–9]. In Germany, a high prevalence of vitamin D deficiency, especially in winter, has been described [10, 11]. Since Germany is at high latitude (47–55° north, Berlin: 52° 31′ N, 13° 24′ E), sun exposure from October to March is not sufficient to maintain an adequate vitamin D supply leading to decreased 25(OH) D levels during winter if no supplements are used [12]. For subjects with darker skin pigmentation, sun-avoiding behavior or a long clothing style this geographical location can be an aggravating factor [13–15]. Already in 1961, Dunnigan et al. described cases of rickets and osteomalacia in the Pakistani Community in Glasgow, Scotland [16]. In the 1970s, the first German case reports about osteomalacia in Turkish guest workers in Germany as consequence of a vitamin D deficiency were published [17, 18].

It is known that non-western immigrants migrating to a country with limited sun exposure are at higher risk for vitamin D deficiency, however there are still considerable knowledge gaps regarding factors other than darker skin or veiling behavior [15, 19–23]. The aim of the present study was therefore to examine and compare the vitamin D status and potentially associated sociodemographic, lifestyle and medical factors in Berliners with and without Turkish roots.

## Methods

### Study design

The present cross-sectional analysis combined two samples from the pre-test phase of the German National Cohort Study (GNC) [24]. The first sample was derived from the “P1 project”, a feasibility study from November 2011 until May 2012, aiming to improve study recruitment among Berliners of Turkish descent [25]. The second sample was a control group recruited from April to May 2012 among an unselected sample of Berliners, excluding those with Turkish roots.

### Recruitment strategy and participants

Details of the recruitment strategy of the “P1 project” were published by Reiss et al. [25]. Briefly, half of the P1 participants and the whole control sample were recruited as a random sample drawn from the residents’ registration office, whereas the other half of the P1 participants were recruited via Turkish networks in Berlin. General practitioners all over Berlin were informed about the study.

For the first recruitment strategy, randomly chosen persons received an invitation letter including study information and a response form. Up to two reminder letters were sent out in case of non-response. If a phone number was available, potential participants were contacted by phone.

To identify persons with Turkish origin, an onomastic procedure (i.e. selection of individuals based on the origin of their first or family names) was applied for the “P1 project” sample by the Robert Koch-Institute, the national Public Health institute in Germany. Potential participants received all study material in both the German and Turkish language. Bilingual study assistants, mostly medical students, conducted up to three home visits on different days and times. If a potential participant was not at home, an information card with contact details was left at the home address.

For the community-based recruitment approach, key persons in Turkish networks were invited to provide information about the study and to participate in focus groups. Bilingual study staff distributed study materials in migrant settings (shops, doctor’s offices, religious and educational institutions, and societies) and invited people to the study.

Inclusion criteria were 20–69 years of age, the ability to understand the aims of the study, and a signed informed consent (in German or Turkish). All participants received a 15€ expense allowance to cover travelling costs and a letter with results from the study center visit by post.

The study was approved by the ethical review committee of the Charité-Universitätsmedizin Berlin, Germany.

### Data collection

#### Questionnaire

The modified health questionnaire from the German Health Interview and Examination Survey was used to assess socioeconomic status, medical history, health behavior, physical and mental diseases and migration background [26]. A participant was defined as being of Turkish descent if (i) he/she or (ii) at least one parent was born in Turkey [27]. Participants with Turkish roots could choose between self-completed questionnaire versions in the German or Turkish language. In case of difficulties understanding the questions, dyslexia (i.e. reading difficulties) or presbyopia (i.e. age-related reduced ability to focus on near objects), bilingual staff was present in the study center to conduct the questionnaire as a face-to-face interview. Among the control sample the questionnaire was applied as an interview by trained study staff according to standard operating procedures (SOP).

### Measures

#### Physical examinations

In the “P1 project”, weight, height, circumference of hip and waist, blood pressure and heart rate were measured by trained physicians and nurses according to SOPs; in the control sample, weight and height were assessed by self-report.

#### Blood analyses

A blood sample was taken by trained physicians or nurses according to SOPs. The blood samples were analyzed for 25(OH) D, full blood count, liver enzymes, kidney function, cholesterol levels, and blood glucose. Vitamin D was measured as 25(OH) D in serum via chemiluminescence immunoassay by the “hospital-laborverbund Brandenburg-Berlin”. Participants received their results as postal letter two weeks after their visit in our study center.

#### Covariates

Physical activity was dichotomized into categories of 150 min or more vs less than 150 min physical activity per week according to WHO recommendations [28]. Body mass index (BMI) was calculated with normal weight being defined as a BMI between greater than 18.5 and less than 25 kg/m^2^, overweight as BMI from 25 to less than 30 kg/m^2^ and obesity as a BMI of 30 kg/m^2^ and higher. Alcohol consumption was categorized into no consumption, moderate consumption (up to 12 grams/day (g/d) for women and up to 24 g/d for men) and hazardous consumption (> 12 g/d for women and > 24 g/d for men). Smoking was dichotomized in never or ex-smoker versus smoker. A person was defined as a smoker, if he or she indicated smoking daily or occasionally, while ex-smokers were defined as former smokers who quit smoking at least one year ago.

### Statistical analysis

We computed descriptive statistics for sociodemographic, lifestyle and medical characteristics using mean with standard deviation (SD) and median for continuous variables and absolute and relative frequencies for categorical variables.

Potential influences on the serum vitamin D concentration as the primary outcome were evaluated using univariable and multivariable linear regression models that were stratified for study sample. Means with 95% confidence intervals (95% CI) are presented from univariable regression in the subgroup of women with Turkish background. We used a stepwise backwards approach for the selection of the co-variables in the multivariable linear regression model with a limit for inclusion of *p* < 0.2. In the final model for the sample with Turkish background the variables BMI, physical activity and sex were included. In the final model for the sample without Turkish background, BMI and sex were included. Resulting Beta-estimates are presented with 95%-CI and *p*-value.

As a sensitivity analysis we conducted for each study sample stratified univariable and multivariable logistic regression analyses with the binary outcome variable “vitamin D deficiency” based on the recommendations by the Institute of Medicine [29]. Crude odds ratios resulting from univariable logistic regression were estimated for the factors mentioned above. In the multivariable logistic regression model, we used a stepwise backwards approach for the selection of the co-variables with a limit for inclusion of *p* < 0.2. In the final model for the sample with Turkish background the variables age, physical activity and chronic disease were included. All odds ratios are presented with 95%-CIs. For the sample without Turkish background the variables sex, BMI, physical activity, smoking status and chronic disease were included.

All analyses were explorative rather than strictly hypotheses testing. Missing data were not imputed. All analyses were performed using The SAS System®, Version 9.4 under Windows operating system.

## Results

**Fig. 1:**
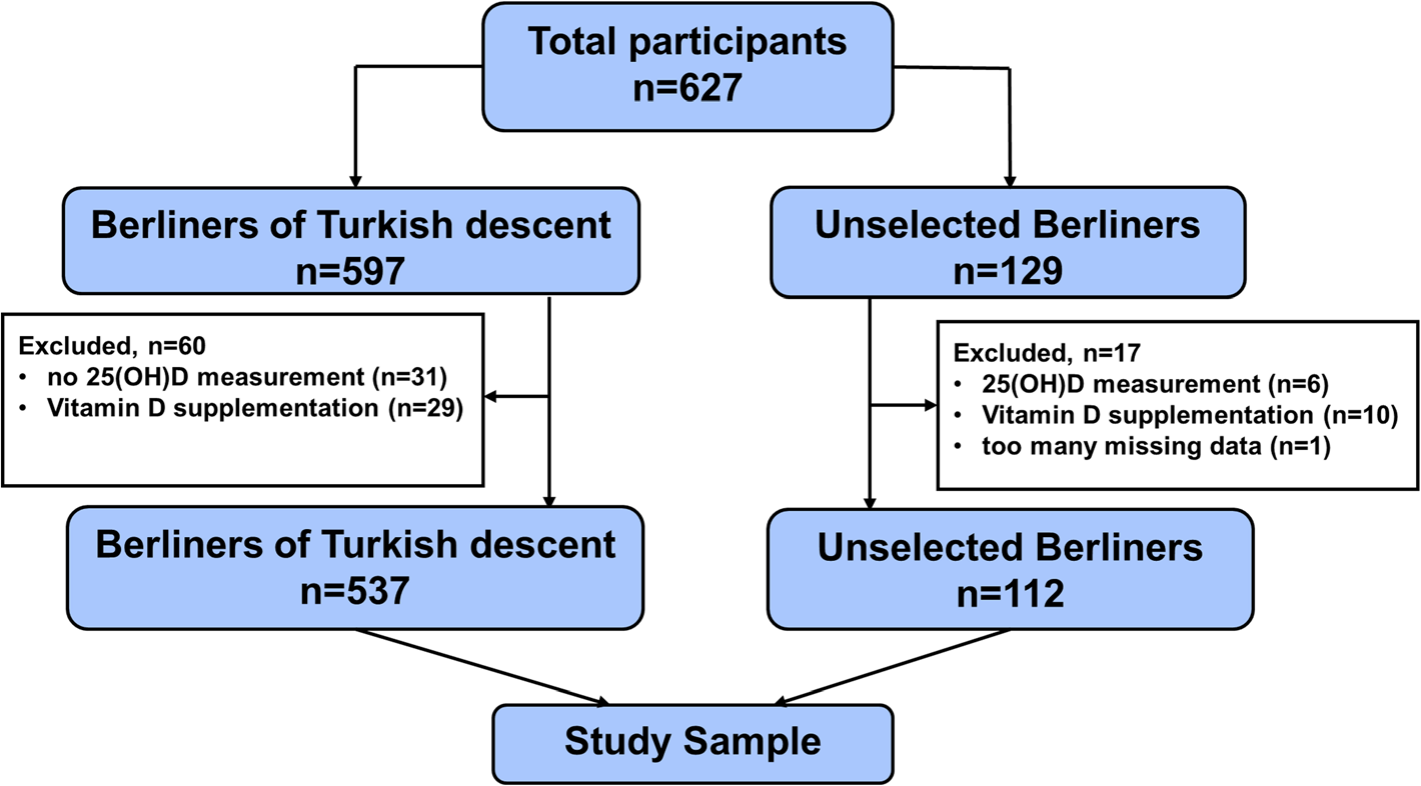
STROBE recruitment flowchart

The study included 597 persons of Turkish descent and a random sample of 129 Berliners without Turkish roots. After excluding participants without vitamin D measurement, with regular vitamin D intake, and those with a high percentage of missing data the final analysis sample consisted of 537 persons with Turkish background and 112 unselected Berliners without such a background as the control group (see Fig. 1 for the STROBE flowchart).

### Characteristics of the study sample and vitamin D status

**Fig. 2:**
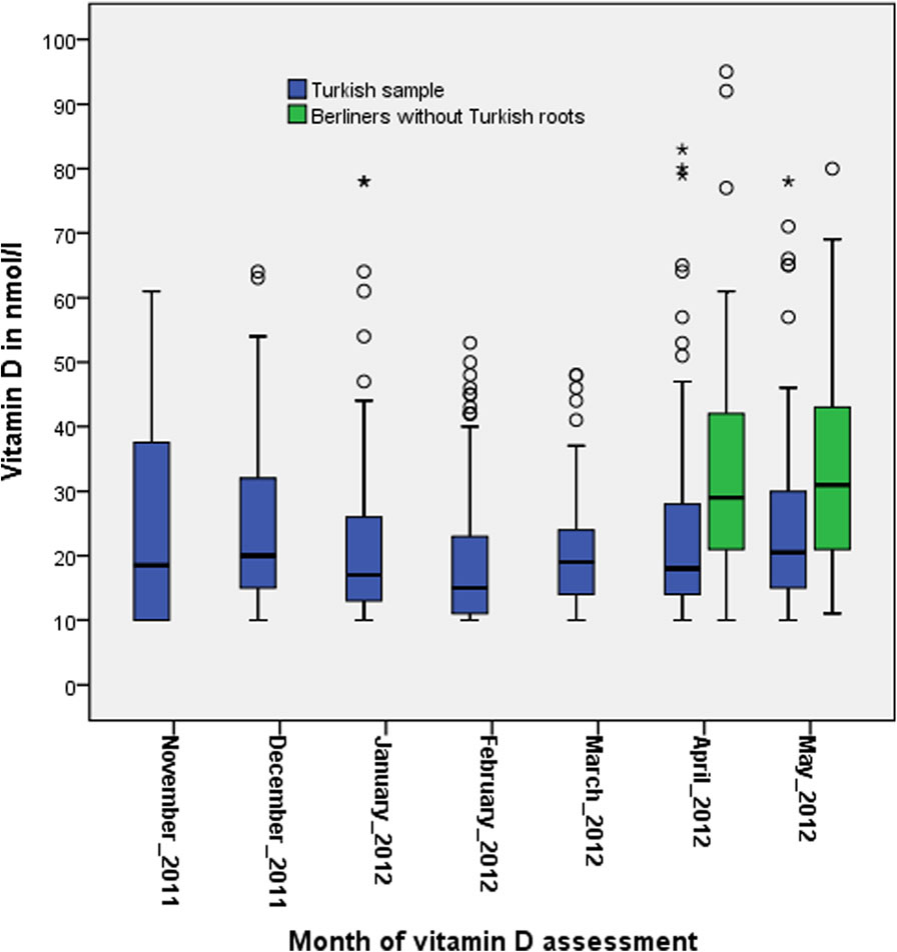
Changes in vitamin D serum levels during the assessment period in both study samples

Among the Turkish sample BMI was higher whilst educational level, physical activity, and alcohol consumption were lower, and women had more often at least one chronic disease than the sample of unselected Berliner women without Turkish background. Considerable differences were observed for the serum concentration of vitamin D. Stratified by sex, the mean 25(OH) D level was lower in the Turkish sample: 22.5 ± 13.8 nmol/L vs 31.5 ± 13.8 nmol/L among women (*p* = 0.001), and 22.9 ± 13.0 nmol/L vs 38.5 ± 26.7 nmol/L among men (*p* = 0.002). In the Turkish sample 94.8% of the women and 95.2% of the men had vitamin D levels below 50 nmol/L (suggesting “vitamin D deficiency” [29]) vs 86.9% (women) and 78.4% (men) among the unselected Berlin sample (both with *p* < 0.001). Vitamin D concentrations of under 25 nmol/L (suggesting “severe vitamin D deficiency” [29]) could be observed in 70.3% vs 36.1% among women and 66.2% vs 35.3% among men when comparing the Turkish sample with the unselected sample without Turkish roots; *p* < 0.001. For details see Table 1. The analysis of the subsample of women with Turkish background showed an even lower mean 25(OH) D concentration for veiled women: 14.4 nmol/L (11.5;17.3) vs 24.9 nmol/L (23.1;26.7), (p < 0.001) in unveiled women. The average vitamin D concentration changed slightly over the assessment period (seven months in the Turkish sample and two months in the sample without Turkish roots) reflecting seasonal sun exposure. See Fig. 2 and Additional file 1: Table S1.

**Table 1:** Characteristics of the two study samples

	Women		Men	
	Berliners of Turkish descent *N* = 330	Unselected Berliners without Turkish roots *N* = 61	Berliners of Turkish descent *N* = 207	Unselected Berliners without Turkish roots *N* = 51
Sociodemographics	% (n) or mean ± SD			
Age (in years)	42.7 ± 12.9	44.4 ± 14.7	44.0 ± 11.6	49.4 ± 14.2
BMI (kg/m^2^)^a^	29.0 ± 6.1	24.5 ± 4.3	29.0 ± 4.6	25.9 ± 3.4
Education in years	9.7 ± 2.6	11.9 ± 1.6	10.1 ± 2.3	11.6 ± 1.8
Education categories				
< 10 years	43.4 (121)	8.2 (5)	37.1 (72)	15.7 (8)
10–12 years	37.3 (104)	24.6 (15)	42.3 (82)	23.5 (12)
> 12 years	19.4 (54)	67.2 (41)	20.6 (40)	60.8 (31)
Lifestyle				
Physical activity				
≥ 150 min/d	12.1 (40)	45.9 (28)	11.6 (24)	52.9 (27)
< 150 min/d	87.9 (290)	54.1 (33)	88.4 (183)	47.1 (24)
Smoking status				
never- or ex-smoker	56.3 (178)	65.6 (40)	53.2 (108)	51.0 (26)
current smoker	43.7 (138)	34.4 (21)	46.8 (95)	49.0 (25)
Alcohol consumption				
none (0 g/d)	64.1 (177)	9.8 (6)	36.7 (69)	9.8 (5)
moderate (> 0-12 g/d for women, > 0-24 g/d for men)	35.1 (97)	83.6 (51)	62.2 (117)	72.5 (37)
hazardous (> 12 g/d for women, > 24 g/d for men)	0.7 (2)	6.6 (4)	1.1 (2)	17.6 (9)
Veiling (only women)			–	
scarf and long clothes in summer	16.7 (43)	–	–	–
scarf but no long clothes	7.0 (18)	–	–	–
long clothes in summer but no scarf	3.1 (8)	–	–	–
no	73.2 (188)	–	–	–
Self-report of diagnosed diseases				
Osteoporosis	8.6 (22)	0	0.6 (1)	0
Thyroid disease	32.7 (93)	16.4 (10)	5.8 (10)	3.9 (2)
At least one cardiovascular disease^b^	33.6 (111)	11.5 (7)	27.5 (57)	39.2 (20)
Diabetes	15.3 (42)	3.3 (2)	12.4 (22)	0
At least one chronic disease^c^	58.8 (174)	29.5 (18)	39.3 (72)	39.2 (20)
Serum concentrations				
Vitamin D 25(OH) D serum concentration in nmol/L	22.5 ± 13.8	31.5 ± 13.8	22.9 ± 13.0	38.5 ± 26.7
25(OH) D Status^d^				
< 50 nmol/L (“deficiency”)	94.8 (313)	86.9 (53)	95.2 (197)	78.4 (40)
25- < 50 nmol/L (“moderate deficiency”)	24.5 (81)	50.8 (31)	29.0 (60)	43.1 (22)
< 25 nmol/L (“severe deficiency”)	70.3 (232)	36.1 (22)	66.2 (137)	35.3 (18)
50–75 nmol/L (“insufficiency”)	3.9 (13)	13.1 (8)	3.9 (8)	11.8 (6)
> 75 nmol/L (“sufficiency”)	1.2 (4)	0 (0)	1.0 (2)	9.8 (5)
Calcium (mmol/l)	2.3 ± 0.1	2.3 ± 0.1	2.3 ± 0.1	2.3 ± 0.1

SD, standard deviation

^a^Height and weight were assessed by measurement among the Berliners of Turkish descent, while it was self-report among the unselected Berliners without Turkish descent

^b^Hypertension, coronary heart disease (CHD), myocardial infarction, heart insufficiency, peripheral arterial disease (PAD) or stroke

^c^At least one out of the diseases mentioned in this table

^d^Vitamin D status according to the Institute of Medicine of the National Academies (IOM (Institute of Medicine) 2011)

In the sample of Berliners of Turkish descent, persons who were obese, with low physical activity, who did not smoke, did not have a chronic disease and women who were veiled more often had vitamin D levels < 50 nmol/L; all *p* < 0.05 (Additional file 1: Tables S2 and S5). Among the unselected sample without Turkish roots women and non- or ex-smokers were more likely to have vitamin D values < 50 nmol/L; all *p* < 0.05. However trends could be observed also among the unselected sample for an association of obesity and low physical activity with a higher probability of a vitamin D concentration < 50 nmol/L (Additional file 1: Tables S3 and S6).

### Factors associated with serum vitamin D levels

Univariable and multivariable linear regression analyses (stratified for both study samples) revealed that among the sample with Turkish background a low physical activity level, being overweight and obese were independently associated with lower vitamin D levels (Table 2). Among the unselected sample of Berliners without Turkish background, again being overweight and obese, but also female sex, was associated with lower vitamin D levels (however, the association with female sex was only borderline significant with *p* = 0.059, considering the conventional significance level of *p* < 0.05) (Table 3). A first analysis indicated that hazardous alcohol consumption was associated with higher vitamin D levels. On closer examination however it was revealed that this was caused by one outlier with an extremely high vitamin D concentration and also very high alcohol consumption. Due to the small sample size we examined the effect of this one individual closer and conducted the analysis with and without this subject. We present the analysis as Table S4 in our Additional file 1.

**Table 2:** Vitamin D concentration as the outcome based on univariable and multivariable linear regression analyses (beta-estimates with 95% confidence levels) – sample with Turkish background

Effect (Vitamin D level)				
	Univariable Analysis		Multivariable Analysis^a^	
	Beta Estimates	p	Beta Estimates *n* = 530	p
Age (linear variable) (N = 537)	−0.1	0.035	*Not included in final model*	
Sex: male vs. female (N = 537)	0.4	0.721	0.9	0.452
Education: < 10 years vs ≥10 years (*N* = 473)	−2.2	0.078	*Not included in final model*	
BMI: normal weight vs. overweight/obesity (N = 530)	3.8	0.005	3.7	0.005
Physical activity: ≥150 min/d vs < 150 min/d (N = 537)	5.0	0.006	4.8	0.008
Smoking Status: never−/ex-smokers vs. smokers (*N* = 519)	−1.22	0.308	*Not included in final model*	
Chronic disease: at least one vs none (*N* = 479)	−1.3	0.29	*Not included in final model*	
Alcohol consumption: never or moderate vs. hazardous (*N* = 464)	−7.26	0.30	*Not included in final model*	

^a^Stepwise backward elimination (based on *p* = 0.2)

**Table 3:** Vitamin D concentration as the outcome based on univariable and multivariable linear regression analyses (beta-estimates with 95% confidence levels) – unselected sample without Turkish background

Effect (Vitamin D level)				
	Univariable Analysis		Multivariable Analysis^a^	
Variable Name (N used for univariable Model)	Beta Estimates	p	Beta Estimates *n* = 109	p
Age (linear variable) (*N* = 111)	−0.05	0.64	*Not included in final model*	
Sex: male vs. female (N = 111)	4.5	0.165	6.2	0.059
Education: < 10 years vs ≥10 years (*N* = 111)	−4.5	0.392	*Not included in final model*	
BMI: normal weight vs. overweight/obesity (*N* = 109)	6.6	0.05	7.8	0.018
Physical activity: ≥150 min/d vs < 150 min/d (*N* = 111)	4.5	0.164	*Not included in final model*	
Smoking Status: never−/ex-smokers vs. smokers (*N* = 111)	−1.8	0.589	*Not included in final model*	
Chronic disease: at least one vs none (*N* = 111)	−1.0	0.779	*Not included in final model*	
Alcohol consumption: never or moderate vs. hazardous (*N* = 111)	−1.4	0.782	*Not included in final model*	

^a^Stepwise backward elimination (based on *p* = 0.2)

Additionally, we conducted a logistic regression analysis as sensitivity analysis with vitamin D deficiency as the outcome stratified for both study samples (Additional file 1: Table S5 and S6).

## Discussion

Our study showed that vitamin D levels were significantly lower among Berliners of Turkish descent, and especially in veiled women, compared to a control sample of the local Berlin population without Turkish roots.

This observation is in line with previous studies, where the proportion of low vitamin D levels and vitamin D deficiency in children, adolescents and adults with migration background was found to be considerably higher than in the existing population of the country that they migrated to [15, 18, 30–32]. Erkal et al. reported average 25(OH)D-values of 38.1 nmol/L for persons with Turkish background and 68.5 nmol/L for a German control group [15], while our results, which were even lower, confirm this gap. If differentiated in low (< 50 nmol/L) and very low (< 25 nmol/L) vitamin D concentrations, our results emphasize that migrant populations show more often very low vitamin D levels (often defined as severe deficiency) than the general local non-migrant population as also reported by Tarner et al. [21].

Besides the known risk factor of darker skin among many migrants with Turkish background, the fact that Berlin is a metropolitan area with limited access to green spaces in large parts of the inner city, could also be a reason for the high percentage of low and very low vitamin D levels [19]. Our results are in line with several studies showing that wearing long clothing is an important risk factor for low vitamin D levels among Turkish women in Turkey [13, 33] and especially in northern and central Europe, as shown in a previous German study that was conducted in the Ruhr region [34].

Other risk factors besides migration background also play a role in vitamin D levels. However, study results regarding the determinants vary [19]. When comparing our results with previously published studies, only some of the determinants had similar effects on vitamin D levels, namely low physical activity, high BMI, and sex. This was shown by other authors, who also found that education and age were associated with low vitamin D levels, which is in contrast to our findings [10, 35, 36]. We found relatively similar associations in both study samples. Among both samples, a high BMI was significantly associated with lower vitamin D levels, which can partly be explained by the repository effects of adipose tissue. A further explanation for reduced vitamin D levels is the higher sedentary behavior and more indoor activities among persons with higher BMI [37]. In the Turkish sample, a low physical activity was associated with lower vitamin D levels, which is in line with other study results; physical activity has been shown to be associated with vitamin D levels, the mechanism however is not clear. While some studies interpret physical activity as a proxy of sunlight exposure, other studies report effects of physical activity irrespective of whether it is performed indoors or outdoors [35, 37]. A factor that was associated with vitamin D in the unselected sample was smoking. In contrast to other studies, we observed a higher percentage of vitamin D deficiency, defined as concentrations <50nmo/L, in non- or ex-smokers than in smokers. This association may be explained partly by the fact that smokers often go outside to smoke (and are therefore more exposed to sunlight) since the introduction of a smoking ban that prohibits smoking inside public buildings [35]. In the linear regression analyses however, we did not find an association of smoking with the vitamin D concentration.

Only in the unselected sample was female sex associated with lower vitamin D levels. This is in contrast to other German studies, where no sex-specific differences were observed [10, 22]. Although the findings of the rather small sample should not be over interpreted, one possible explanation could be more distinct lifestyle differences between men and women in that sample than in the Turkish sample. For instance men engaged more often in physical activity and were more often smokers. Previous studies showed correlations between vitamin D deficiency and cardiovascular diseases [3, 38]. We assessed self-reported diagnoses of several major chronic diseases but were not able to confirm any associations between cardiovascular or other chronic diseases such as diabetes or osteoporosis with the vitamin D concentration.

One of the strengths of our study was the considerable effort including home visits that was made to recruit participants with a Turkish migration background. We reached a participation rate of over 10% which is much higher than in other population-based studies without any sub-group-specific activities to increase the response [25, 39]. Another strength was the use of stringent epidemiological assessment methods by using questions from the national German health surveys with translations into Turkish. Furthermore, the bilingual questionnaires and staff in the recruitment office, during home visits and in the study center contributed to the relative recruitment success by allowing participants to use the language they felt most comfortable with, thus increasing general acceptance and trust in the aims of the study.

However, certain limitations of our study need to be considered such as the relatively small control sample of unselected Berliners without Turkish roots, which may have reduced the statistical power for rather rare exposures. Another limitation is that “home leave” was not assessed among the Turkish sample. Holidays in a sunny country could have increased the annual mean vitamin D concentration and low vitamin D levels during winter could have been compensated by that. Further, no eating habits were assessed and therefore nutritional information of the participants are lacking. A third limitation is the relatively short assessment period which allowed us to collect blood only in winter, spring and early summer where vitamin D levels are generally lower than at the end of summer. So we cannot present the course of vitamin D levels over a full year and all 4 seasons. We observed small seasonal changes with the lowest vitamin D concentrations from January through March which is in line with another German study of Turkish migrants [40]. However, these seasonal changes during the assessment period are in our opinion unlikely to have produced a seasonal bias or distorted our study results.

## Conclusions

Serum vitamin D levels were considerably low in Berliners of Turkish descent, and especially in veiled women. Factors associated with low vitamin D levels were high BMI and low physical activity in the sample with Turkish background and high BMI and female sex in the unselected sample.

Possible future public health actions include weight reduction programs and physical activity promotion for the development of preventive strategies regarding a sufficient vitamin D status, especially in Berliners with Turkish background. Further research is needed to identify other potential public health strategies.

## Additional file

Additional file 1: **Table S1.** Changes in vitamin D serum levels during the assessment period in both study samples. **Table S2.** Vitamin D Status of Berliners of Turkish descent (*n* = 537) by potentially associated factors. **Table S3.** Vitamin D status of Berliner sample without Turkish roots (*n* = 112) by potentially associated factors. **Table S4.** Vitamin D concentration as the outcome based on univariable and multivariable linear regression analyses (beta-estimates with 95% confidence levels) – unselected sample without Turkish background (including one participant with outlier: Vitamin D of 164 nmol/L). **Table S5.** Vitamin D deficiency as outcome based on univariable and multivariable logistic regression analyses (odds ratios with 95% confidence levels) – sample with Turkish background. **Table S6.** Vitamin D deficiency as the outcome based on univariable and multivariable logistic regression analyses (odds ratios with 95% confidence levels) – unselected sample without Turkish background. (DOCX 28 kb)

## Abbreviations

25(OH)-D3: 25-Hydroxy-Vitamin-D3, Calcidiol
BMI: Body mass index
CI: Confidence interval
GNC: German National Cohort Study
PTH: Parathormone
SD: Standard deviation
SOP: Standard operating procedure
UVBR: Ultraviolet B radiation
