# Supplementary material for: Serum vitamin D levels in Berliners of Turkish descent –a cross-sectional study
Source: BMC Public Health. 2019 Jan 28;19:119. doi: 10.1186/s12889-019-6446-5 (PMC6350357; doi:10.1186/s12889-019-6446-5)
Supplement: Supplementary file 1 — Table S1. Changes in vitamin D serum levels during the assessment period in both study samples. Table S2. Vitamin D Status of Berliners of Turkish descent (n = 537) by potentially associated factors. Table S3. Vitamin D status of Berliner sample without Turkish roots (n = 112) by potentially associated factors. Table S4. Vitamin D concentration as the outcome based on univariable and multivariable linear regression analyses (beta-estimates with 95% confidence levels) – unselected sample without Turkish background (including one participant with outlier: Vitamin D of 164 nmol/L). Table S5. Vitamin D deficiency as outcome based on univariable and multivariable logistic regression analyses (odds ratios with 95% confidence levels) – sample with Turkish background. Table S6. Vitamin D deficiency as the outcome based on univariable and multivariable logistic regression analyses (odds ratios with 95% confidence levels) – unselected sample without Turkish background. (DOCX 28 kb) [file 12889_2019_6446_MOESM1_ESM.docx]

**Table S1** Changes in vitamin D serum levels during the assessment period in both study samples

|  | Berliners of Turkish descent | | | Berliners without Turkish roots | | |
| --- | --- | --- | --- | --- | --- | --- |
| Month | Vitamin D in nmol/L  Mean±SD (Median) | Vitamin D deficiency in % | n | Vitamin D in nmol/L  Mean±SD (Median) | Vitamin D deficiency in % | n |
| November 2011 | 24.3±16.8 (18.5) | 91.7 | 12 | - | - | - |
| December 2011 | 25.4±14.3 (20.0) | 90.6 | 53 | - | - | - |
| January 2012 | 22.5±15.4 (17.0) | 92.9 | 70 | - | - | - |
| February 2012 | 19.1±11.0 (15.0) | 98.2 | 110 | - | - | - |
| March 2012 | 20.8±9.5 (19.0) | 100.0 | 74 | - | - | - |
| April 2012 | 24.3±15.3 (18.0) | 92.7 | 110 | 35.1±23.8 (29.0) | 83.3 | 66 |
| May 2012 | 24.5±13.7 (20.5) | 94.4 | 108 | 34.1±16.0 (31.0) | 82.6 | 46 |

**Table S2** Vitamin D status of Berliners of Turkish descent (n=537) by potentially associated factors

| Sociodemographic, medical and lifestyle factors | 25(OH)D Status^a^  % (n) | | | p-value |
| --- | --- | --- | --- | --- |
|  | <50nmol/L  95.0 (510) | 50-75nmol/L  3.9 (21) | >75nmol/L  1.1 (6) |  |
| Age categories |  |  |  | 0.209 |
| >42 years | 96.0 (266) | 3.6 (10) | 0.4 (1) |  |
| ≤42 years | 93.8 (244) | 4.2 (11) | 1.9 (5) |  |
| Sex |  |  |  | 0.965 |
| Men | 95.2 (197) | 3.9 (8) | 1.0 (2) |  |
| Women | 94.8 (313) | 3.9 (13) | 1.2 (4) |  |
| Veiled^b^ |  |  |  | 0.121 |
| yes | 100 (69) | 0 | 0 |  |
| no | 94.1 (177) | 4.3 (8) | 1.6 (3) |  |
| Education |  |  |  | 0.948 |
| <10 years | 94.8 (183) | 4.1 (8) | 1.0 (2) |  |
| 10-12 years | 95.7 (178) | 3.2 (6) | 1.1 (2) |  |
| >12 years | 93.6 (88) | 5.3 (5) | 1.1 (1) |  |
| BMI |  |  |  | 0.031 |
| normal | 92.9 (130) | 4.3 (6) | 2.9 (4) |  |
| overweight | 92.9 (169) | 6.0 (11) | 1.1 (2) |  |
| obese | 98.1 (204) | 1.9 (4) | 0 |  |
| Self-report of doctor diagnosed diseases |  |  |  |  |
| Osteoporosis |  |  |  | 0.460 |
| yes | 91.3 (21) | 4.3 (1) | 4.3 (1) |  |
| no | 95.0 (384) | 3.7 (15) | 1.2 (5) |  |
| [Thyroid](https://www.dict.cc/englisch-deutsch/thyroid.html) [disease](https://www.dict.cc/englisch-deutsch/disease.html) |  |  |  | 0.410 |
| yes | 96.1 (99) | 3.9 (4) | 0 |  |
| no | 94.6 (333) | 3.7 (13) | 1.7 (6) |  |
| Diabetes |  |  |  | 0.557 |
| yes | 96.9 (62) | 3.1 (2) | 0 |  |
| no | 94.3 (365) | 4.1 (16) | 1.6 (6) |  |
| At least one cardiovascular disease |  |  |  | 0.196 |
| yes | 93.5 (157) | 6.0 (10) | 0.6 (1) |  |
| no | 95.7 (353) | 3.0 (11) | 1.4 (5) |  |
| At least one chronic disease  (one out of all diseases above) |  |  |  | 0.049 |
| yes | 94.3 (232) | 5.3 (13) | 0.4 (1) |  |
| no | 95.7 (223) | 2.1 (5) | 2.1 (5) |  |
| Physical activity |  |  |  | 0.008 |
| ≥150 min/d | 89.1 (57) | 6.3 (4) | 4.7 (3) |  |
| <150 min/d | 95.8 (453) | 3.6 (17) | 0.6 (3) |  |
| Smoking status |  |  |  | 0.024 |
| never- or ex-smoker | 96.2 (275) | 3.8 (11) | 0 |  |
| smoker | 93.6 (218) | 3.9 (9) | 2.6 (6) |  |
| Alcohol consumption |  |  |  | 0.104 |
| moderate or never | 94.8 (436) | 3.9 (18) | 1.3 (6) |  |
| hazardous | 75.0 (3) | 25.0 (1) | 0 |  |

a: Vitamin D status according to IOM (IOM (Institute of Medicine) 2011); b: scarf, long clothes in summer or both**Table S3** Vitamin D status of Berliner sample without Turkish roots (n=112) by potentially associated factors

| Sociodemographic, medical and lifestyle factors | 25(OH)D Status^a^  % (n) | | | p-value |
| --- | --- | --- | --- | --- |
|  | <50nmol/L 83.0 (93) | 50-75nmol/L 12.5 (14) | >75nmol/L  4.5 (5) |  |
| Age categories |  |  |  | 0.776 |
| ≤42 years | 84.6 (33) | 12.8 (5) | 2.6 (1) |  |
| >42 years | 82.2 (60) | 12.3 (9) | 5.5 (4) |  |
| Sex |  |  |  | 0.044 |
| Men | 78.4 (40) | 11.8 (6) | 9.8 (5) |  |
| Women | 86.9 (53) | 13.1 (8) | 0 (0) |  |
| Education |  |  |  | 0.284 |
| <10 years | 69.2 (9) | 23.1 (3) | 7.7 (1) |  |
| 10-12 years | 81.5 (22) | 18.5 (5) | 0 |  |
| >12 years | 86.1 (62) | 8.3 (6) | 5.6 (4) |  |
| BMI |  |  |  | 0.743 |
| normal | 80.0 (48) | 13.3 (8) | 6.7 (4) |  |
| overweight | 83.8 (31) | 13.5 (5) | 2.7 (1) |  |
| obese | 92.3 (12) | 7.7 (1) | 0 |  |
| Self-report of doctor diagnosed diseases |  |  |  |  |
| Osteoporosis |  |  |  | - |
| yes | 0 | 0 | 0 |  |
| no | 83.0 (93) | 12.5 (14) | 4.5 (5) |  |
| [Thyroid](https://www.dict.cc/englisch-deutsch/thyroid.html) [disease](https://www.dict.cc/englisch-deutsch/disease.html) |  |  |  | 0.253 |
| yes | 100.0 (12) | 0 | 0 |  |
| no | 81.0 (81) | 14.0 (14) | 5.0 (5) |  |
| Diabetes |  |  |  | 0.590 |
| yes | 50.0 (1) | 50.0 (1) |  |  |
| no | 83.6 (92) | 11.8 (13) | 4.5 (5) |  |
| At least one cardiovascular disease |  |  |  | 0.214 |
| yes | 74.1 (20) | 22.2 (6) | 3.7 (1) |  |
| no | 85.9 (73) | 9.4 (8) | 4.7 (4) |  |
| At least one chronic disease  (one out of all diseases above) |  |  |  | 0.339 |
| yes | 78.9 (30) | 18.4 (7) | 2.6 (1) |  |
| no | 85.1 (63) | 9.5 (7) | 5.4 (4) |  |
| Physical activity |  |  |  | 0.151 |
| ≥150 min/d | 76.4 (42) | 16.4 (9) | 7.3 (4) |  |
| <150 min/d | 89.5 (51) | 8.8 (5) | 1.8 (1) |  |
| Smoking status |  |  |  | 0.047 |
| never- or ex-smoker | 89.4 (59) | 6.1 (4) | 4.5 (3) |  |
| smoker | 73.9 (34) | 21.7 (10) | 4.3 (2) |  |
| Alcohol consumption |  |  |  | 0.118 |
| moderate or never | 83.8 (83) | 13.1 (13) | 3.0 (3) |  |
| hazardous | 76.9 (10) | 7.7 (1) | 15.4 (2) |  |

## a: Vitamin D status according to IOM (IOM (Institute of Medicine) 2011)

**Table S4** Vitamin D concentration as the outcome based on univariable and multivariable linear regression analyses (beta-estimates with 95% confidence levels) – unselected sample without Turkish background (including one participant with outlier: Vitamin D of 164nmol/L)

| Effect (Vitamin D level) | | | | | |
| --- | --- | --- | --- | --- | --- |
|  | Univariable Analysis | | Multivariable Analysis^a^ | | |
| Variable Name (N used for univariable Model) | Beta Estimates | p | Beta Estimates  n=110 | | p |
| Age (linear variable) (N=112) | 0.07 | 0.63 | *Not included in final model* | | |
| Sex: male vs. female (N=112) | 7.0 | 0.077 | 6.7 | | 0.100 |
| Education:  <10 years vs ≥10 years (N=112) | 5.9 | 0.341 | *Not included in final model* | | |
| BMI: normal weight vs. overweight/obesity (N=110) | 3.9 | 0.336 | 6.8 | | 0.100 |
| Physical activity:  ≥150min/d vs <150min/d (N=112) | 6.8 | 0.084 | 6.9 | | 0.080 |
| Smoking Status: never-/ex-smokers vs. smokers (N=112) | 0.22 | 0.957 | *Not included in final model* | | |
| Chronic disease: at least one vs none (N=112) | -2.7 | 0.517 | *Not included in final model* | | |
| Alcohol consumption: never or moderate vs. hazardous (N=112) | -11.38 | 0.064 | -12.7 | 0.044 | |

**Table S5** Vitamin D deficiency as the outcome based on univariable and multivariable logistic regression analyses (odds ratios with 95% confidence levels) – sample with Turkish background

| **Effect (Vitamin D deficiency: <50nmol/L)** | | |
| --- | --- | --- |
|  | **Univariable Analysis** | **Multivariable Analysis^a^** |
|  | **Crude Odds Ratios**  **[95%CI]** | **adjusted Odds Ratios [95%CI], n=370** |
| Age (linear variable) | 1.0 [1.0;1.08] | 1.1[1.0;1.1] |
| Sex: female vs. male (N=537) | 0.9 [0.4;2.1] | *Not included in final model* |
| Education:  ≥10 years vs. <10 years (N=473) | 1.0 [0.5;2.4] | *Not included in final model* |
| BMI: Overweight/obesity vs. normal weight (N=530) | 1.7 [0.8;3.8] | *Not included in final model* |
| Physical activity:  <150min/d vs. ≥150min/d (N=537) | 2.8 [1.1;6.9] | 2.1 [0.7;5.8] |
| Smoking Status: Smokers vs. never-/ex-smokers | 0.6 [0.3;1.3] | *Not included in final model* |
| Chronic disease: at least one vs. none (N=479) | 0.7 [0.3;1.7] | 0.5 [0.2;1.3] |
| Alcohol consumption:  never or moderate vs. risky (N=464) | 6.1 [0.6;60.4] | *Not included in final model* |

a: Stepwise backward elimination (based on p=0.2)

**Table S6** Vitamin D deficiency as the outcome based on univariable and multivariable logistic regression analyses (odds ratios with 95% confidence levels) – unselected sample without Turkish background

| **Effect (Vitamin D deficiency: <50nmol/L)** | | |
| --- | --- | --- |
|  | **Univariable Analysis** | **Multivariable Analysis^a^** |
|  | **Crude Odds Ratios**  **[95%CI]** | **Adjusted Odds Ratios [95%CI], n=110** |
| Age (linear variable) (N=112) | 1.0 [0.96;1.03] | *Not included in final model* |
| Sex: female vs. male (N=112) | 1.8 [0.67;4.95] | *Not included in final model* |
| Education:  ≥10 years vs. <10 years (N=112) | 2.5 [0.68;9.13] | *Not included in final model* |
| BMI: Overweight/ obesity vs.  normal weight (N=110) | 1.5 [0.6;4.3] | 2.4 [0.7;7.9] |
| Physical activity:  <150min/d vs. ≥150min/d (N=112) | 2.63 [0.9;7.5] | 2.8 [0.9;8.5] |
| Smoking Status:  Smokers vs. never-/ex-smokers (N=112) | 0.3 [0.1;0.9] | 0.3 [0.1;0.9] |
| Chronic disease: at least one vs. none (N=112) | 0.7 [0.2;1.8] | 0.4 [0.1;1.3] |
| Alcohol consumption:  never or moderate vs. risky (N=112) | 1.6 [0.4;6.3] | *Not included in final model* |

a: Stepwise backward elimination (based on p=0.2)
